# Addressing Challenges to Enhance the Bioactives of* Withania somnifera* through Organ, Tissue, and Cell Culture Based Approaches

**DOI:** 10.1155/2017/3278494

**Published:** 2017-02-16

**Authors:** Pritika Singh, Rupam Guleri, Amrita Angurala, Kuldeep Kaur, Kulwinder Kaur, Sunil C. Kaul, Renu Wadhwa, Pratap Kumar Pati

**Affiliations:** ^1^Department of Biotechnology, Guru Nanak Dev University, Amritsar, Punjab 143 005, India; ^2^Drug Discovery and Assets Innovation Lab, DBT-AIST International Laboratory for Advanced Biomedicine (DAILAB), Biomedical Research Institute, National Institute of Advanced Industrial Science & Technology (AIST), Tsukuba 305 8565, Japan

## Abstract

*Withania somnifera* is a highly valued medicinal plant in traditional home medicine and is known for a wide range of bioactivities. Its commercial cultivation is adversely affected by poor seed viability and germination. Infestation by various pests and pathogens, survival under unfavourable environmental conditions, narrow genetic base, and meager information regarding biosynthesis of secondary metabolites are some of the other existing challenges in the crop. Biotechnological interventions through organ, tissue, and cell culture provide promising options for addressing some of these issues.* In vitro* propagation facilitates conservation and sustainable utilization of the existing germplasms and broadening the genetic base. It would also provide means for efficient and rapid mass propagation of elite chemotypes and generating uniform plant material round the year for experimentation and industrial applications. The potential of* in vitro* cell/organ cultures for the production of therapeutically valuable compounds and their large-scale production in bioreactors has received significant attention in recent years.* In vitro* culture system further provides distinct advantage for studying various cellular and molecular processes leading to secondary metabolite accumulation and their regulation. Engineering plants through genetic transformation and development of hairy root culture system are powerful strategies for modulation of secondary metabolites. The present review highlights the developments and sketches current scenario in this field.

## 1. Introduction

*Withania somnifera* (L.) Dunal (family Solanaceae) holds high repute in Indian, Greek, and African traditional system of medicine [1]. It is commonly referred to as Ashwagandha, Asgandh, Winter cherry, or Indian ginseng. It is present in World Health Organization (WHO) monographs on most important medicinal plants [2] and has been included in the list of top thirty-two prime concerned medicinal plants by the National Medicinal Plant Board of India (http://www.nmpb.nic.in) owing to its huge demand in both domestic and international markets [3]. The plant is implicated in prevention and cure of a range of ailments such as cancer, arthritis, asthma, aging, inflammation, and neurological disorders [1]. The pharmacological activities are attributed to occurrence of diverse secondary metabolites which includes alkaloids, flavanol glycosides, glycowithanolides, steroidal lactones (withanolides), sterols, and phenolics [4–6]. The distribution of these pharmaceutically important metabolites is found in the aerial parts, roots, and berries of* W. somnifera* [2].

*W. somnifera* is predominantly propagated through seeds [7]. Its traditional cultivation has been limited due to low percentage of seed viability, poor germination and seedling survival [8], and low yield of withanolides from natural sources. In addition, infestation with pathogens and pests [9, 10] and a narrow genetic base pose a serious challenge in its cultivation and improvement. Recently, a huge interest is generated for enhancement of pharmacological important metabolite content of this important medicinal plant through biotechnological interventions [1]. In this context, judicious use of tools which could provide an opportunity for the conservation, utilization, and creation of genetic diversity is much warranted. Furthermore, integrated approaches involving new molecular tools and available biological resources will pave the way for enhancement of commercial value of the crop.

The concentration of pharmacologically important withanolides in* W. somnifera* is quite low, ranging from 0.001 to 0.5% of dry weight [2]. Further, chemical synthesis of withanolides is cumbersome and there is lack of information on biosynthesis and regulation of secondary metabolites. In this regard, cell/organ cultures hold an immense potential for mass production of secondary metabolites. Cell suspensions usually synthesize metabolites at a faster rate owing to their uniform and active growth. Moreover, cell suspension cultures can be used as a tool to understand various plant physiological processes. Apart from cell/organ cultures, genetic transformation (both* Agrobacterium tumefaciens* and* A. rhizogenes* mediated) has emerged as a powerful tool for engineering the plants for overexpressing desired metabolites and to decipher molecular functions of selected genes. The objective of the present review is to highlight the biotechnological interventions to enhance the secondary metabolite production in* W. somnifera*.

## 2. *In Vitro* Propagation

### 2.1. Micropropagation

In medicinal plants, production of a large number of elite chemotypes without seasonal constraints is highly desirable.* In vitro* technique such as micropropagation could be of special use for their rapid multiplication as well as for generating uniform quality clones. Shoot multiplication studies in* W. somnifera* have been carried out by several workers using different explants (Table 1).

Most of the studies in* W. somnifera* have documented the use of Murashige and Skoog's (MS) medium [11] for micropropagation and seed germination. However, other media such as Schenk and Hildebrandt (SH) [12], B5 [13], WPM [14], and J. P. Nitsch and C. Nitsch (NN) [15] have also been used for shoot multiplication [16–18]. Studies have shown that the seed germination largely depends on type of plant growth regulator (PGR), temperature, and light. Among various PGRs [IAA, IBA, 2,4-D, BAP, and GA], maximum percentage of seed germination was obtained with GA_3_ (433 *µ*M) at 25°C [19]. Different carbon sources have also been tested for* in vitro* propagation. Normally, sucrose (2-3%) is employed for shoot proliferation and rooting [18]. Increasing the sucrose concentration (4–10%) resulted in reduced growth of shoots, whereas fructose (2%) led to lesser proliferation of microshoots. The use of glucose however did not improve rate of shoot multiplication and rooting as compared to sucrose [16]. Furthermore,* in vitro* flowering and fruit set formation was found to be influenced by carbon and nitrogen sources in elongated shoots of* W. somnifera* [20]. Besides, appropriate physical parameters such as light, temperature, and relative humidity have also been established to play crucial role in* in vitro* propagation. Photoperiod of 14–16 h per day under 30–45 *µ*mol/m^2^/s provided by cool, white fluorescent lamps and the temperature set at 25 ± 2°C have been found to be suitable for culture incubation.

**Figure 1:**
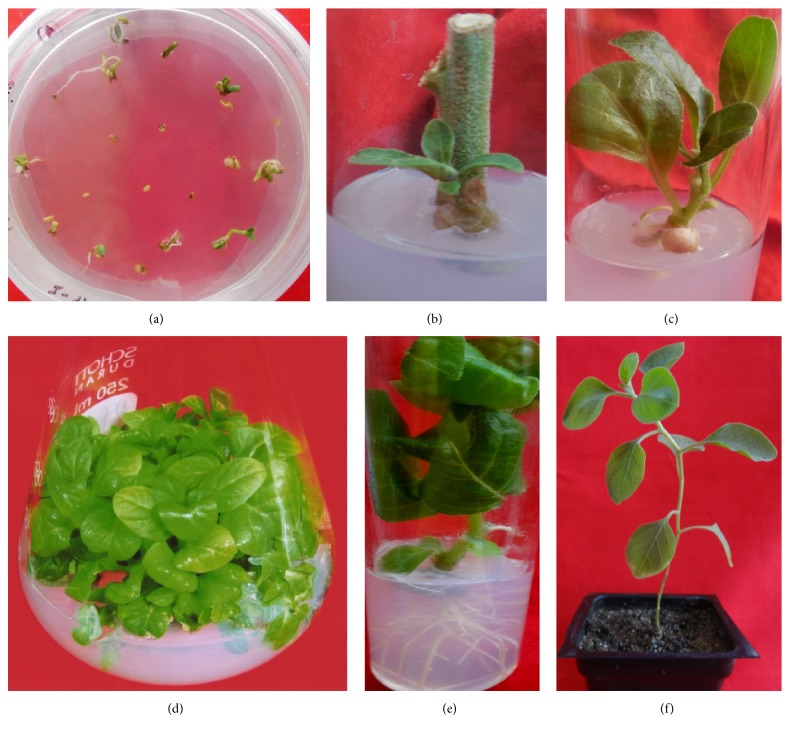
Micropropagation of* W. somnifera*. (a) Initiation of aseptic cultures from seeds; (b) nodal explants in MS basal medium; (c) shoots growing in BAP (5 *µ*M); (d) shoot proliferation; (e) rooted microshoots in IBA (10 *µ*M); (f) hardened plant.

The proliferation and growth of microshoots and their rooting largely depend on the exogenous application of PGRs. Research reports indicate that BAP is most effective for multiple shoot formation and proliferation. BAP (2–9 *µ*M) alone or in conjunction with IAA (2–11 *µ*M) has been commonly used for multiple shoot formation depending upon the nature of explant [16, 18, 21, 22]. MS medium fortified with BAP (2.5–5 *µ*M) is being used for shoot proliferation in micropropagation protocol developed in our laboratory (Figure 1) [23]. However, combinations of BAP and KN [24] and BAP and IBA [17, 25] have also been tried for shoot formation and their multiplication. Recent findings suggest that addition of inorganic nutrients such as CuSO_4_ (100 *µ*M) and ZnSO_4_ (300 *µ*M) [26] and polyamines (spermidine 20 mg/l) [27] in plant growth medium results in enhanced shoot proliferation. Zinc is required for synthesis of tryptophan, a precursor for auxin synthesis. Addition of ZnSO_4_ in the nutrient medium could elevate the level of auxin* via* tryptophan, resulting in shoot proliferation [28]. Cu and Zn also constitute essential components of various enzymes which play an important role in cellular metabolic processes, such as protein synthesis, carbohydrate synthesis, and electron transport [29, 30]. Furthermore, studies have shown that reducing the agar concentration to half increases the proliferation rate of shoots [16]. Shoots grown in liquid MS medium containing BAP (4.44 *µ*M) showed enhanced shoot multiplication. A twofold increase in shoot growth was reported in liquid medium amended with 10% (v/v) coconut milk [16]. In another study, liquid culture medium having BAP (2.66 *µ*M) along with spermidine (20 mg/l) was found to be suitable for shoot multiplication [31]. However, hyperhydricity of shoots is one of the major limitation of liquid culture system in* W. somnifera* [32].

Rooting of microshoots has been successfully achieved in both MS and half-strength MS medium with and without PGRs. Among various auxins employed for* in vitro* rooting, IBA (5–10 *µ*M) was found to be most suitable [18, 21, 33, 34]. Study conducted in author's laboratory also shows that IBA (10 *µ*M) is best suited for rooting both in terms of biomass and plant growth (Figure 1) [23]. IAA (2-3 *µ*M) alone or in combination with IBA has also been used for rooting [22, 35, 36]. It has been observed that rooting is inhibited by high concentrations of auxins leading to callus formations at the base [37]. Microshoots thus rooted are used for successful hardening and establishment of plantlets under field conditions. Usually, mixture of sand and soil (1 : 1) is employed for hardening [22, 38, 39] but vermiculite and manure have also been tried along with sand-soil mix [18, 21, 27, 40]. Genetic fidelity of multiple shoots generated from cotyledonary nodes has been analyzed using different markers like RAPD and ISSR [41]. RAPD technique has also been used to identify somaclonal mutant among the regenerated* W. somnifera* plantlets overproducing 12-deoxywithastramonolide [42].

Although there are many reports on* in vitro* propagation of* W. somnifera* using various explants, the fine tuning of existing protocols and optimization of microenvironment is essential for establishing a rapid and efficient propagation system for this high value medicinal plant. Efforts should also be directed to develop cost effective and mass production of genetically uniform elite chemotype of* W. somnifera* round the year for subsequent experimentation and industrial applications.

### 2.2. Regeneration Studies

An efficient and reproducible plant regeneration system is imperative for numerous applications such as genetic manipulations, variant selection, and transformation. Regeneration studies in* W. somnifera* have been summarized in Tables 2–4.

#### 2.2.1. Shoot Bud Regeneration

Selection of explant is a crucial factor in shoot bud regeneration. Various explants such as leaf, stem, epicotyl, node, internode, axillary shoot tip, petiole, hypocotyl, root, cotyledonary leaf, and embryo are employed for regeneration (Tables 2 and 3). However, leaves and nodal segments have been most commonly used for shoot bud regeneration. It is observed that very young leaves or very old leaves of* W. somnifera* fail to regenerate due to the developmental stage specific auxin distribution in the plant [38]. Likewise, the regeneration potential of petiole explant was found to be lower than that of leaves [33]. Among the* in vitro* and* in vivo* explants, it was observed that* in vivo* leaves were better suited for callus mediated regeneration compared to* in vitro* leaves [37]. The differential response of explants is governed by their physiological status, as* in vitro* conditions such as nutrient medium and hormones have been reported to influence the nuclear and cellular behavior of tissue culture generated plants [43, 44]. Comparison of different explants shows that regeneration frequency from the callus is the highest in leaf followed by cotyledon, hypocotyl and epicotyl [45]. Calli obtained from hypocotyl were most favourable for shoot regeneration as compared to those generated from root and cotyledonary leaf segments [46]. The culture vessels were also shown to play an important role in regeneration. Culture tubes were found to be better suited for regeneration than Petri dishes [38]. This could be attributed to high concentration of ethylene present in the culture tubes. It was further observed that the regeneration mainly occurs from the surface of leaf directly in contact with the media irrespective of its orientation (adaxial or abaxial) [38].

**Figure 2:**
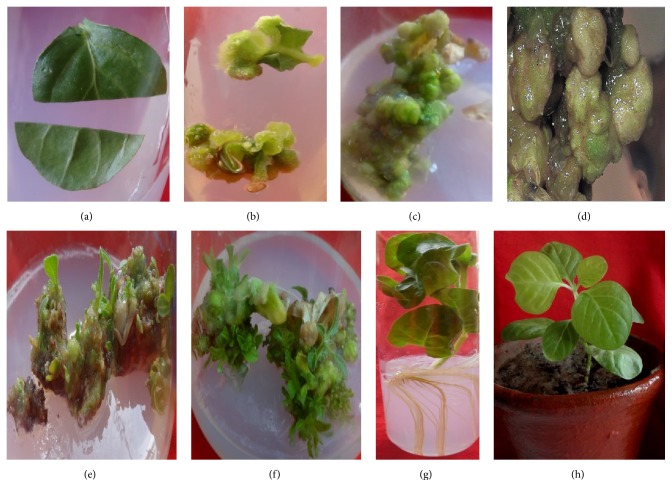
Regeneration from* W. somnifera* leaf explants. (a) Leaf explant from* in vitro* shoots; (b) initiation of callus formation in BAP (15 *µ*M); (c) shoot bud formation in the same medium; (d) shoot buds observed under stereomicroscope; (e) emergence of shoots in BAP (5 *µ*M); (f) proliferation of shoots in BAP (5 *µ*M); (g) rooting of microshoots in IBA (10 *µ*M); (h) hardening of microshoots.

Cell differentiation and organogenesis are regulated by interplay of auxins and cytokinins. Normally, BAP (2–9 *µ*M) alone leads to regeneration via shoot bud formation but its combination with IAA (1–8 *µ*M) has a more pronounced effect on shoot regeneration and multiplication [34, 38, 47]. BAP has also been used in combination with KN [48] and NAA [33, 49]. We have also standardized a regeneration protocol from leaf explant employing BAP (15 *µ*M) (Figure 2) [23]. In several reports, TDZ has been shown to have a role in regeneration [47, 50]. This may be attributed to the ability of TDZ to modulate the endogenous level of growth regulators [51]. Regeneration via callus formation was achieved by fortifying the medium with various combinations of 2,4-D (3–14 *µ*M) and KN (1–9 *µ*M) depending upon the explant (Table 3) [34, 37, 46, 52, 53]. Although a combination of 2,4-D and KN is most commonly used for callus induction, some workers have also used BAP (4–9 *µ*M) along with IAA (2.85 *µ*M) and 2,4-D (4.5 *µ*M) for induction of callus [37, 54]. High concentrations of IAA (56 *µ*M) and KN (56 *µ*M) was used to induce callus from shoot tips and leaves [55]. After the callus induction, BAP (4–9 *µ*M) alone or in conjunction with IAA (0.5 *µ*M) or NAA (5.37 *µ*M) was found to be most effective in maintenance of calli and shoot regeneration [18, 37, 46, 47, 53].

#### 2.2.2. Somatic Embryogenesis

There are only few records of somatic embryogenesis in* W. somnifera* (Table 4). Moreover, these reports are not very convincing. Calli obtained from cotyledonary leaf segments and internodal segments developed into somatic embryos when cultured on medium containing KN (13.95 *µ*M) and BAP (8.88 *µ*M) along with TIBA (4.0 *µ*M), respectively. Embryos formed differentiated into shoots when cultured on medium containing BAP (4.4 *µ*M) and IAA (11.4 *µ*M) [56]. In another report, leaf calli cultured on medium containing 2,4-D (2.26 *µ*M) and BAP (4.44 *µ*M) along with casein hydrolysate (10 mg/l) also induced somatic embryos in* W. somnifera* [57].

It is realized that a reproducible regeneration system with high efficiency is much warranted for improvement of* W. somnifera*. It will also facilitate in functional validation of genes which might play critical role in the enhancement of its commercial value. Till now a limited number of explants have been tried for direct and indirect regeneration with a low rate of success. New strategies with different explants and manipulation of extrinsic and intrinsic factors could be useful in this direction.

## 3. Modulation of Secondary Metabolites Using* In Vitro* Culture System

### 3.1. Organ and Callus Cultures

*In vitro* cultures have been effectively used as a model system to study the production and accumulation of secondary metabolites in* W. somnifera*. The* in vitro* system possesses the capacity for higher production and accumulation of metabolites owing to the active growth and higher rate of metabolism within a short period. Withaferin A and withanone content was documented to be significantly higher in tissues of* in vitro* raised* W. somnifera* plants as compared to the field-grown parent plants [27]. A respective increase of 1.14-fold and 1.20-fold was recorded in the content of withaferin A and withanones in leaves of* in vitro* derived plants as compared to field plants, whereas roots of the former exhibited 1.10-fold higher accumulation of withanolide A than the latter. In another study,* in vitro* regenerated plants showed twofold higher withaferin A and withanolide A and tenfold increase in withanone contents as compared to field-grown plants [58]. Similar to above reports, a marked increase in withanolide A production was observed in* in vitro* shoot cultures [59]. In the above background, it appears relevant to utilize* in vitro* propagation system as an alternative system for production of pharmacologically important withanolides throughout the year, which are otherwise severely limited in production under natural conditions.

Production of secondary metabolites in* in vitro* system is regulated by media formulations, sucrose concentration, and type and concentration of PGRs. Shoot tips of* W. somnifera* proliferating on B5 medium accumulated maximum withaferin A (0.09%), while withanolide D accumulation was maximal (0.065%) in hormone free medium [16]. Among different media tested (MS, B5, NN, and Chu's N6), half-strength MS medium was found to be optimum for biomass accumulation as well as withanolide A production in adventitious root cultures [60]. Moreover, sucrose concentration in media was directly proportional to withaferin A and withanolide D accumulation. It was observed that accumulation of withaferin A and withanolide D was enhanced significantly when media were supplemented with 4% sucrose without adversely affecting growth [16]. In a recent report, different types and concentration of nitrogen sources (adenine sulphate, L-glutamine, potassium nitrate ammonium nitrate, and sodium nitrate) and various carbon sources (sucrose, glucose, maltose, and fructose) were tried to enhance multiplication of shoots and withanolides accumulation [20]. It was observed that BAP (6.66 *µ*M) and IAA (1.71 *µ*M) with 20 mg/l L-glutamine and 4% sucrose improved the multiple shoot formation whereas change of sucrose to 6%, induced maximum withanolides content. At low concentration (4% in this case), sucrose acts as a carbon source and signaling molecules [61, 62] resulting in optimum biomass accumulation, whereas for secondary metabolite production a high level of sucrose (6% in this case) is considered to be obligatory, as sucrose at higher concentration creates osmotic stress in the medium, enhancing the production of secondary metabolites [63].

The hormonal combinations also modulate the biogenesis of withanolides in the* in vitro* cultures of* W. somnifera*. Shoot cultures produced from shoot tips in MS medium fortified with BAP (4.44 *µ*M) accumulated withaferin A (0.04%) and withanolide D (0.06%) [16]. However, further increase in BAP concentration leads to reduction in withanolide content. Shoots grown on MS medium containing BAP (4.44 *µ*M) and KN (2.32 *µ*M) showed higher accumulation of withanolide A (0.24%) [64]. In a recent report it was suggested that shoots cultured in half-strength MS liquid medium amended with BAP (5.0 *µ*M) accumulated higher biomass and withaferin A (1.5-fold) within 5 weeks of culture [65]. The enhanced accumulation of biomass and withaferin A due to BAP may attribute to its property to induce shoot proliferation [66], as withaferin A is present majorly in the leaves [1]. It was also observed that various glycowithanolides (Physagulin D, Withastraronolide, Withanoside IV, and Withanoside VI) accumulate in shoots cultured in BAP (4.44 *µ*M) [67]. Studies also indicate that adventitious roots produced directly from leaf segments on half-strength MS medium having IBA (2.46 *µ*M) showed higher accumulation of withanolide A (21-fold) compared to leaves of natural plants [60]. Similarly, adventitious roots upon elicitation by biotic elicitor, chitosan (100 mg/l) for 4 h stimulated higher production of all withanolides [68]. PGRs also had a significant influence on the secondary metabolites production in callus. The friable callus derived from shoot tips, nodes, and leaf segments of* in vitro* germinated seedlings of* W. somnifera* in media containing 2,4-D and KN did not contain withanolide A and withaferin A, whereas solid callus induced in media supplemented with IBA and BAP had both of these withanolides [52]. High levels of withanolides such as withanolide A and withanolide B were also recorded in* in vitro* fruits. However, withaferin A was found to accumulate more in* in vitro* flowers as compared to fruits, whereas no significant difference was observed in withanone accumulation in both* in vitro* flowers and fruits [20]. The differential/organ specific accumulation of secondary metabolites may attributable to tissue specific regulation of its synthesis [58, 69].

In addition to above factors, supplementing media with casein hydrolysate (500 mg/l) in liquid system favoured withanolide D synthesis (0.10%), whereas addition of coconut milk 10% (v/v) favoured biomass accumulation (27-fold) and enhanced withaferin A synthesis (0.136%) [16]. The shoot cultures of* W. somnifera* on liquid MS medium fortified with BAP (2.6 *µ*M) and spermidine (20 mg/l) showed increased amount of withanolides: 6-fold (withanolide A), 7.6-fold (withanolide B), and 1.12-fold (withaferin A) [31]. Enhanced production of secondary metabolites could be attributed to the fact that polyamines together with PGRs activate specific genes involved in biosynthesis of secondary metabolites [27]. Exogenous application of elicitors in culture system is one of the most promising approaches for enhancing the production of valuable secondary metabolites. Among the different abiotic elicitors, MeJ and SA have been most commonly used for the induction of secondary metabolites [31]. Multiple shoot cultures of* W. somnifera* grown in liquid medium and elicited with MeJ (100 *µ*M) and SA (100 *µ*M) for 4 h led to enhanced production of withanolide A (14-fold and 16-fold), withanolide B (11-fold and 13-fold), withaferin A (13-fold and 15-fold), and withanone (12-fold and 14-fold), respectively, when compared to control [31]. Comparison of MeJ and SA shows that elicitation by SA was significantly higher than that by MeJ. Adventitious root cultures derived from leaf also resulted in increased production of withanolide A (48-fold), withanolide B (29-fold), withaferin A (20-fold), withanone (37-fold), 12-deoxywithastramonolide (9-fold), withanoside V (7-fold), and withanoside IV (9-fold) when treated with SA (150 *µ*M) for 4 h [70].

### 3.2. Cell Suspension Culture

In medicinal plants, cell suspension cultures have been extensively used for producing secondary metabolites [71]. In* W. somnifera* also plant cell suspension culture holds great promise, as it could be successfully used for enhancement of withanolides as well as to study the cellular and molecular processes leading to the secondary metabolites accumulation. Normally, a relatively less yield of withanolides especially, withanolide A and withaferin A is obtained in cell suspension cultures [72–74]. To enhance withanolide production in cell suspension cultures, various factors such as media formulations, carbon source, PGRs, macro- and micronutrient compositions, and inoculum mass have been explored [74, 75]. Among different media, MS medium was recorded to be best suited for both biomass accumulation and withanolide A production [74]. The other factor which has a profound effect on the withanolide production is the type and concentration of carbon source. Sucrose (2–4%) was found to be optimum for the biomass accumulation as well as for the withanolide A production in comparison to glucose, fructose, and maltose. The possible explanation for this might be that sucrose upon hydrolysis produces glucose, which is readily utilized for cell growth [74, 75]. Enhancement in withanolide content could also be achieved by manipulating medium salt concentrations. The ratio of ammonia/nitrate ions and total nitrogen content significantly affected the production of secondary metabolites, making nitrogen nutrient an excellent candidate to manipulate during medium optimization. In high concentration, ammonium becomes toxic if it is not metabolized immediately, thus exerting inhibitory effect on cellular metabolism. NO_3_^−^ is first reduced to NH_4_^+^ before being incorporated into amino acids [76, 77]. The cellular homeostasis of ammonium is maintained by AMT-type transporters. AMT-type transporters are the predominant path for the influx of ammonium and therefore these transporters are tightly regulated at transcription and translation level to prevent cellular toxicity [78]. Under* in vitro* conditions, explants are exposed to ammonium nitrate in the nutrient medium. The tolerance level of explants towards ammonium may correspond to species specific activity of AMT-type transporters. It was observed that higher NO_3_ and lower NH_4_ concentration favour both cell growth and withanolide production [74]. The production of withanolide A was highest in MS medium containing 2.0x KNO_3_ in the cell suspension cultures of* W. somnifera*. Further, highest withanolide A content was shown when ratio of NH_4_^+^/NO_3_^−^ ions was set at 14.38/37.60 mM [73]. Twofold increase in strength of CaCl_2_ concentration also resulted in enhanced withanolide A production [74]. A similar effect of CaCl_2_ has also been documented in a study on suspension culture of* Datura innoxia*, where a tenfold increase in intracellular accumulation of scopolamine and hyoscyamine was observed by the addition of CaCl_2_ [79]. This effect of calcium chloride may be attributed to its dual property to work as abiotic elicitor and secondary messenger.

Role of different PGRs in accumulation of secondary metabolites in cell suspension cultures has also been studied in* W. somnifera*. MS medium containing 2,4-D (9.05 *µ*M) and KN (2.32 *µ*M) showed highest accumulation of biomass and withanolide A production in cell suspension cultures [74]. Similarly, addition of picloram (4.14 *µ*M) in the growth medium led to higher accumulation of withanolides [75]. Besides these parameters, agitation speed also has direct bearing on accumulation of biomass and withanolide production in cell suspensions. Of the different agitation speeds (80 to 160 rpm) tried, the highest biomass accumulation and withanolide production were achieved at 120 rpm [75]. In addition, elicitation is a key strategy to enhance the production and accumulation of pharmaceutically important metabolites in cell suspensions. Ions (calcium chloride and copper sulphate), abiotic agents (arachidonic acid, MeJ, and SA), biotic factors such as plant cell wall components, and other components from a microbiological origin act as potent elicitors. Cell suspension culture of* W. somnifera* produced maximum withaferin A when treated with 100 *µ*M of CuSO_4_ and 5% (v/v)* Verticillium dahliae* cell extract [72]. About 1.45–1.58-fold higher amounts of withanolides were produced by multiple shoots grown in liquid MS medium supplemented with 40%* Gracilaria edulis* extract [80]. A recent study indicates that higher amounts of total withanolides were obtained in shake flask culture (2.13-fold) and bioreactor (1.66-fold) as compared to control, when treated with chitosan (100 mg/l) and squalene (6 mM) along with picloram (1 mg/l), KN (2.32 *µ*M), L-glutamine (200 mg/l), and sucrose (5%) [81]. Such media manipulation strategies could be effective for mass production of secondary metabolites in* W. somnifera*.

### 3.3. Biotransformation

The interconversion of compounds through biotransformation using cell suspension cultures is a promising approach for obtaining desired phytomolecules. Biotransformation by interconversion of withanolides could provide vital information on biosynthesis of important withanolides. Cell suspension cultures obtained from leaves of* W. somnifera* were used for the biotransformation of withanolides to different withanolides and some new compounds [82]. Withaferin A, withanone, and withanolide A were fed to the synchronously growing cell suspension cultures and it was observed that the interconversion of withanolide A to withanone was most significant whereas the interconversion in the opposite route occurs at low levels. This biotransformation mechanism probably involves substitution of hydroxyl group at C-20 position in withanolide A to C-17 in withanone [82]. Identification of genes implicated in position-specific hydroxylation such as cytochrome P450 would further enhance our understanding on biotransformation of withanolides.

## 4. Genetic Manipulation Studies

### 4.1. *Agrobacterium tumefaciens* Mediated Transformation

Development of an efficient transformation system is a prerequisite to understand metabolic pathways and their regulation and to engineer them for specific metabolites. This technique has been successful in a number of important crop plants; however, such efforts are limited in the area of medicinal plants. Transformation studies conducted in* W. somnifera* have been summarized in Table 5. One of the earliest attempts to obtain transformed cultures was made by infecting leaves with wild-type nopaline and octopine strains of* A. tumefaciens* [83]. However, this resulted in production of only shooty teratomas which were able to grow in basal medium.* A. tumefaciens* (LBA4404) harbouring pIG121Hm vector was for the first time used for successful transformation of leaves of* W. somnifera* [84]. Infected leaves were grown in MS medium containing BAP (8.9 *µ*M) and IAA (8.0 *µ*M) along with augmentin (400 mg/l) and selection was made after 2 cycles with kanamycin (50 mg/1). Shoots produced were rooted and successfully acclimatized leading to development of normal fertile* W. somnifera* transgenic plants [84]. Transgenic plants were also regenerated from nodal explants infected with* A. tumefaciens* strain EHA105 containing binary vector pGA492 and grown in MS medium fortified with BAP (6.66 *µ*M) and IAA (1.71 *µ*M) [85]. These studies clearly indicate that factors such as germplasm, source of explant,* A. tumefaciens* strain, infection time, duration of cocultivation, and the selection medium have important bearing on transformation efficiency. Apart from these, physical wounding and addition of antinecrotic compounds also influence efficiency of transformation. Recently, it was demonstrated that vacuum infiltration of* A. tumefaciens* solution for 10 min followed by sonication for 10 sec resulted in higher transient GUS expression in* W. somnifera* [86]. They also reported the effectiveness of thiol compounds in cocultivation medium to improve the gene integration. In another study, recombinant cell lines were generated by overexpressing a key sterol biosynthetic pathway gene (*Squalene synthase*) of* W. somnifera* [87]. The transformed cell suspension culture showed significant enhancement in the activity of squalene synthase (4-fold) and withanolide content (2.5-fold) as compared to nontransformed cell line. Recently, transgenic plants overexpressing* squalene synthase* were also generated by transforming apical and nodal segments. The transformed plants showed an upregulation of expression of* squalene synthase* gene to 2–5-fold and total withanolide content up to 1.5–2-fold [88]. Due to much higher synthesis of withanolides in transformed cultures compared to nontransformed cultures, this strategy could also be explored effectively for metabolic engineering.

### 4.2. *Agrobacterium rhizogenes* Mediated Hairy Root Induction

Hairy root culture has emerged as an attractive system for production of secondary metabolites [89]. The hairy roots are obtained by transfer of T-DNA, from root inducing plasmid (pRi) of* A. rhizogenes* to susceptible plant cells [90]. There are a few reports on production of hairy root cultures of* W. somnifera* using* A. rhizogenes* (Table 6). The transformation efficiency is influenced by various parameters like nature of explant,* Agrobacterium* stain, cocultivation medium, duration of cocultivation, and concentration of acetosyringone [91]. Among various explants, cotyledons and leaves show positive response towards infection with* A. rhizogenes* [92, 93]. For successful transformation the level of tissue differentiation, the age of explant, and the delicate nature of the explants are also critical [93]. Modifying the culture conditions for bacterial growth also has a great influence on hairy root induction. Among various media [Luria Burntti (LB), Nutrient broth (NB), Tryptone broth (TB), Yeast Mannitol Broth (YMB), Tricalcium phosphate broth (TPB), and Yeast Extract Peptone (YEP)] tried, YEP was found to be the most suitable for the growth and maintenance of* A. rhizogenes* [93]. The pH of the medium set at 5.8 was best suited for the growth of hairy roots [94]. Among different carbon sources (sucrose, fructose, and maltose), sucrose (3%) was found to be most effective for the growth of transformed roots [94]. Carbon source also plays an important role in differential accumulation of withanolides in hairy roots. It was observed that withaferin A is accumulated in high amounts in hairy roots when grown in medium containing 4% (w/v) sucrose. However, hairy roots produced both withaferin A and withanolide A only in medium supplemented with 3% (w/v) sucrose [95]. Likewise, incorporating 5% (w/v) glucose in the medium led to higher accumulation of withaferin A and withanolide A [95]. The role of macroelements and nitrogen source has also been investigated to monitor their effects on hairy root production and accumulation of withanolides [96]. Maximum biomass was reported in the medium containing 2.0x KH_2_PO_4_ whereas production of withanolide A was highest in the medium with 2.0x of KNO_3_. It was further observed that the moderate concentration of NH_4_ (14.38 mM) along with high concentration of NO_3_ (37.60 mM) led to higher biomass accumulation and withanolide A production compared to control. This observation could be linked to concentration of nitrogen in culture which affects the amount of proteins or amino acid products. Apart from this, hairy roots also show significantly high antioxidant properties. Hairy roots cultured in liquid medium showed high activity in 1,1-diphenyl-2-picrylhydrazyl radical and beta-carotene linoleic acid model system whereas those grown on solid medium showed high antioxidant activity in hydroxyl radical trapping and brain lipid peroxidation assay [97].

Application of elicitors to hairy root cultures could also improve the secondary metabolite production in plant cell/organ culture [98]. A positive correlation between elicitor treatment and withanolide production could be established in* W. somnifera* hairy roots [99]. The optimum concentrations of MeJ and SA which leads to increased withaferin A, withanolide A, and withanone production were found to be 15 *µ*M and 150 *µ*M, respectively. Though elicitation has resulted in enhancement of secondary metabolites, there is a need to understand the exact elicitation mechanism involved in triggering the production of bioactive molecules. Recent work also suggests that induction frequency of hairy roots could be enhanced by subjecting the explants to sonication (15 sec) followed by heat treatment at 41°C for 5 min [100]. The other exciting possibilities into the hairy root system for metabolic engineering could be the identification of key biosynthetic genes or transcription factors controlling their regulation and raising genetically transformed cultures with these genes. In another cultivated species of* Withania*,* W. coagulans*, transformed hairy roots were produced which overproduced squalene synthase* SS1* gene from* A. thaliana* [101]. These engineered hairy roots produced significantly higher amount of withanolide A compared to* in vitro* roots. Hairy roots produced from* W. somnifera* were also cotransformed with *β*-cryptogein, a gene encoding for a fungal elicitor protein [102]. Cryptogein-cotransformed roots did not show any enhancement in withaferin A and withanolide A content as well as in expression of HMGR, FPPS, and SGT genes. However, cotransformed roots displayed a significant increase in activity of enzyme phenylalanine ammonia lyase (PAL), involved in phenylpropanoid pathway. Higher amount of ferulic acid was also found to be accumulated in cotransformed roots compared to normal hairy roots. This indicates a probable metabolic redirection from withanolide biosynthesis towards phenylpropanoid production in cotransformed hairy roots of* W. somnifera* [102].

Though there have been serious efforts for characterization of genes linked to sterol biosynthetic pathway, very limited success has been achieved in mobilization of these genes to the plant and their further evaluation. This is mainly due to the nonreproducible and less efficient regeneration system available for this important medicinal plant. Hence to genetically improve this plant, better regeneration system, greater understanding of withanolide biosynthetic pathway, and adopting genome rather than gene centric approach could be critical. As organized tissue leads to better accumulation of important metabolites in* W. somnifera*, hairy root holds a promising prospect. Developing a rapid proliferating clone and engineering hairy root for higher production of pharmaceutically important secondary metabolites should also be provided due attention.

## 5. Conclusion

*W. somnifera* has attracted a great deal of attention from researchers all over the world due to its immense therapeutic potential. One of the major challenges in a medicinal plant like* W. somnifera* is to enhance the production of important secondary metabolites linked to human health. In this context,* in vitro* culture system not only offers an excellent tool to propagate the plants for rational scientific use but also provides means to enhance its commercial value. The response of organized tissue versus unorganized cells, different media, and regulators have been evaluated for this purpose. However, understanding the reason for better accumulation in organized organs such as shoots and roots compared to unorganized cells like callus and cell suspension and critical factors regulating the key genes of the pathway will provide requisite inputs for appropriate modulation of secondary metabolites in* W. somnifera*. Further, the potential of organ, tissue, and cell culture system is becoming increasingly relevant for generation of knowledge in the area of biosynthesis, regulation, accumulation, and transport of secondary metabolites of this immensely important medicinal plant.

## Figures and Tables

**Table 1:** Shoot multiplication of *W. somnifera*.

Explant	Response	Medium	Number of shoots/explant	Reference
Nodes	Multiple shoots	MS + 4.4 *µ*M BAP + 4.65 *µ*M KN	21.8	[24]
		MS + 4.44 *µ*M BAP	2.6	[102]
		MS + 2.5 *µ*M BAP + 0.5 *µ*M NAA + CuSO_4_ (100 *µ*M)	61.7	[26]
		MS + 2.5 *µ*M BAP + 0.5 *µ*M NAA + ZnSO_4_ (300 *µ*M)	66.1	
		MS + 6.66 *µ*M BAP + 1.71 *µ*M IAA + 20 mg/l spermidine	46.4	[27]

Cotyledonary nodes	Multiple shoots	MS + 4.44 *µ*M BAP	16.93	[41]

Shoot tips	Multiple shoots	MS + 4.44 *µ*M BAP	10.0	[16]
		NN + 4.44 *µ*M BAP + 4.95 *µ*M IBA	120.0	[17]
		MS + 8.88 *µ*M BAP + 11.4 *µ*M IAA	17.0	[21]
		MS + 13.2 *µ*M BAP + 40% coconut water	20–25	[103]
		MS + 4.4 *µ*M BAP + 2.3 *µ*M 2,4-D	120.0	[25]
		MS + 4.4 *µ*M BAP + 2.5 *µ*M IBA	145.0	

Alginate encapsulated shoot tips	Shoot formation	MS	75.0	
		MS + 2.46 *µ*M IBA	87.0	[104]

Axillary buds	Multiple shoots	MS + 8.8 *µ*M BAP + 0.54 *µ*M NAA	12.0	[105]

Apical buds	Multiple shoots	Revised Tobacco medium (RT) + 4.5 *µ*M 2,4-D	13.72	[40]

Seeds	Multiple shoots	MS + 2.66 *µ*M BAP + 2.28 *µ*M IAA	9.9	[22]
		MS + 4.4 *µ*M BAP + 2.3 *µ*M 2,4-D	120.0	[25]

**Table 2:** Direct regeneration in *W. somnifera*.

Explant	Response	Medium	RR, SE	Reference
Leaf	Shoot buds	MS + 4.4 *µ*M BAP + 7.99 *µ*M IAA	63.3 (RR), 9.33 (SE)	[38]
		MS + 13.2 *µ*M BAP + 7.99 *µ*M IAA	70.0 (RR), 13.37 (SE)	[47]
		MS + 4.0 *µ*M BAP + 4.0 *µ*M KN	90 (RR), 12.1 (SE)	[33]
		MS + 8.88 *µ*M BAP;	23.0 (SE)	[106]
		MS + 8.88 *µ*M BAP + 0.53 *µ*M NAA	17.67 (SE)	
		MS + 6.66 *µ*M BAP + 8.55 *µ*M IAA	100 (RR), 68.0 (SE)	

Node	Shoot buds	MS + 2.22 *µ*M BAP	12.5 (SE)	[46]
		MS + 22.2 *µ*M BAP	10.22 (SE)	
		MS + 0.91 *µ*M TDZ	10.1 (SE)	
		MS + 1.36 *µ*M TDZ	12.3 (SE)	
		MS + 2.5 *µ*M BAP + 0.5 *µ*M NAA	95 (RR), 36.1 (SE)	[48]
		MS + 6.66 *µ*M BAP + 1.71 *µ*M IAA	90 (RR), 42.4 (SE)	[84]

Internodes	Shoot buds	MS + 4.44 *µ*M BAP	>25 (SE)	[46]
		MS + 22.2 *µ*M BAP	>40 (SE)	

Epicotyl	Shoot buds	MS + 8.88 *µ*M BAP + 1.14 *µ*M IAA	85 (RR), 15.5 (SE)	[34]

Hypocotyl	Shoot buds	MS + 2.22 *µ*M BAP	12.88 (SE)	[46]

Embryo	Shoot buds	MS + 0.91 *µ*M TDZ	10.0 (SE)	[46]

Shoot tip	Shoot buds	MS + 2.5 *µ*M BAP	92 (RR), 15.1 (SE)	[48]

Petiole	Shoot bud	MS + 8.88 *µ*M BAP	3.67 (SE)	[33]
		MS +8.88 *µ*M BAP + 0.53 *µ*M NAA	2.67 (SE)	

RR: regeneration response (%); SE: shoots/explant.

**Table 3:** Indirect regeneration in *W. somnifera*.

Explant	Response	Medium	C, RR, SE	Reference
Leaf	Callus induction and shoot regeneration	MS + 9.05 *µ*M 2,4-D + 0.93 *µ*M KN	92.0 (C), 8.0 (RR), 3.0 (SE)	[51]
	Callus induction	MS + 56 *µ*M IAA + 56 *µ*M KN	70 (C)	[55]
	Callus induction and shoot regeneration	MS + 8.88 *µ*M BAP + 2.85 *µ*M IAA	92 (C), 89.5 (RR)	[54]
	Callus induction	MS + 1.5 *µ*M IBA	95 (C)	[107]
	Callus induction	MS + 4.44 *µ*M BAP + 4.5 *µ*M 2,4-D	43.4 (C)	[37]
	Shoot regeneration	MS + 8.88 *µ*M BAP + 5.37 *µ*M NAA	82.3 (RR), 4.8 (SE)	
	Callus induction	MS + 13.5 *µ*M 2,4-D	93 (C)	[108]
	Shoot regeneration	MS + 17.6 *µ*M BAP	85 (RR)	
	Callus induction	MS + 2.26 *µ*M 2,4-D + 0.93 *µ*M KN	98.33 (C)	[52]
	Shoot regeneration	MS + 8.88 *µ*M BAP	98.33 (RR)	

Shoot	Callus induction and shoot regeneration	MS + 8.87 *µ*M BAP IAA	84.0 (C), 70.0 (RR), 8.0 (SE)	[51]
	Callus induction	MS + 57 *µ*M	100 (C)	[55]

Node	Callus induction	MS + 4.44 *µ*M BAP + 9.2 *µ*M KN	85 (C)	[109]
	Shoot regeneration	MS + 4.44 *µ*M BAP + 11.6 *µ*M KN	80 (RR), 4.35 (SE)	

Internode	Callus induction	MS + 4.44 *µ*M BAP + 4.5 *µ*M 2,4-D	38.3 (C)	[37]

Cotyledon	Callus induction	MS + 9.05 *µ*M 2,4-D + 0.93 *µ*M KN	100.0 (C)	[39]

Epicotyl	Callus induction	MS + 9.05 *µ*M 2,4-D + 2.79 *µ*M KN	98.3 (C)	[34]
	Shoot regeneration	MS + 4.44 *µ*M BAP + 20 mg/L Adenine sulphate	86.7 (RR), 25.3 (SE)	

Hypocotyl	Callus induction and shoot regeneration	MS + 9.05 *µ*M 2,4-D + 0.93 *µ*M KN	90.0 (C), 44.0 (RR), 4.0 (SE)	[51]
	Callus induction and shoot regeneration	MS + 9.05 *µ*M 2,4-D + 0.93 *µ*M KN	90.7 (C), 44.0 (RR), 4.0 (SE)	[39]

Stem	Callus induction and Shoot regeneration	MS + 2.26 *µ*M 2,4-D MS + 4.44 *µ*M BAP + 0.57 *µ*M IAA	70.0 (RR), 6.2 (SE)	[53]

Root	Callus induction and shoot regeneration	MS + 9.05 *µ*M 2,4-D + 0.93 *µ*M KN	100.0 (C)	[51]
	Callus induction	MS + 9.05 *µ*M 2,4-D + 0.93 *µ*M KN	100.0 (C)	[39]

C: callus induction (%); RR: regeneration response (%); SE: shoots/explant.

**Table 4:** Somatic embryogenesis of *W. somnifera*.

Explant	Medium	Response	Reference
Cotyledonary leaf callus	MS + 13.95 *µ*M KN	Somatic embryogenesis	[56]
Internode and cotyledonary leaf callus	MS + 8.88 *µ*M BAP + 4.0 *µ*M TIBA	Somatic embryogenesis	
Leaf calli	MS + 2.26 *µ*M 2,4 D + 4.44 *µ*M BAP + 10 mg/l casein hydrolysate	Somatic embryogenesis	[57]

**Table 5:** *A*. *tumefaciens *mediated genetic transformation in *W. somnifera*.

Explant	Strain	Transgene cassettes	Response/comments	Reference
Leaf	C58, N2/73, T37, A281, Ach_5_ and disarmed strain LBA 4404	—	Shooty teratomas showed increase in withanolide production	[82]
	LBA 4404	pIG121Hm carry 3 expression cassettes nos:*npt*II:nospA::35S: *GUS-INT*:nospA::35S:*hpt*II:pA	Transformation frequency, 1.67%	[83]
	GV3101	pBWsSS (derived from pBI121) carry 2 expression cassettes nos:*npt*II:nospA::35S:*WsSS*:nospA	Transformation frequency, 70% 4-fold increase in activity of squalene synthase and 2.5-fold enhancement in withanolide A content	[86]

Node	EHA105	pGA492 carry 3 expression cassettes nos:*npt*II:nospA::35S:*GUS-INT*:nospA::35S:*bar*:pA	Transformation frequency, 3.16%	[84]
	LBA 4404	pCAMBIA2301 carry 2 expression cassettes 35S:*npt*II:35SpA::35S:*GUS-INT*:nospA	Transformation frequency, 10% Vacuum infiltration, sonication and addition of thiol compounds resulted in higher transformation efficiency	[85]

Node and apical segment	GV2260	pCAMBIA1301 carry 3 expression cassette 35S:*WsSQS*:nospA::35S: *GUS*:nospA::35S:*hpt*II: nospA	Efficiency of (1) *A. tumefaciens* mediated transformation, 3.86% (2) Microprojectile bombardment, 3.62% (3) Microprojectile bombardment assisted agroinfection, 8.71% 2–5-fold enhancement in *WsSQS* transcript and 1.5–2-fold enhancement in total withanolide	[87]

Seed	MTCC-431	—	Seeds were infected with bacteria for 24 h and then germinated on MS medium containing GA (5.7 *µ*M)	[110]

**Table 6:** *A. rhizogenes *mediated genetic transformation in *W. somnifera.*

Explant	Strain	Response/comment	Reference
Leaf	A4, LBA 9402 and LBA 9360	Strain specificity of the *A. rhizogenes* and its transformation frequency was analyzed. A4 was shown to have higher transformation ability among all the strains tried	[111]
	MTCC 2364, MTCC 532	Transformation efficiency, 20%. The growth rate of hairy root was tenfold more than control	[92]
	LBA 9402, A4	Observed variations in phenotype and withasteroid accumulation of transformed hairy roots	[112]
	R1601	Withanolide A content was 2.7-fold higher in transformed hairy roots compared to control	[91]
	R1601	Withanolide A production was favoured at 4% sucrose and initial medium pH – 6.0 results in highest withanolide A production	[93]
	R1000	Transformation efficiency in petiole 64%, leaf 42.5%, and internode 37.7%. Efficiency was further enhanced to 93.2% by incorporating acetosyringone in different stages of infection	[113]
	R1601	Studying the effect of macroelements and ammonia-nitrate ratio on withanolide A production. Maximum withanolide A production was recorded in ammonia-nitrate ratio of 0.00/18.80 mM	[95]
	R1000	Transformation efficiency, 90%. Acetosyringone (100 *µ*M) was added to bacterial suspension one hour prior to infection. Hairy roots elicited with MeJ or SA showed tremendous increase in withanolide A, withanone and withaferin A content	[98]
	LBA9402	Constitutive expression of fungal elicitor protein, *β*-cryptogein, led to a shift in metabolic flux from withanolide biosynthesis to phenylpropanoid pathway	[101]
	R1000	High transformation efficiency (93.3%) was achieved by sonication and heat treatment	[99]
	R1000/A4	Transformation efficiency 88.4% with R1000 and 79% with A4. High efficiency was obtained by supplementing cocultivation medium with acetosyringone (100 *µ*M)	[90]

Cotyledon	R1601	Withanolide A content was 2.7-fold higher in transformed hairy roots as compared to control	[91]
	R1601	A correlation was made between macroelements, ammonia-nitrate ratio, and withanolide A production. Maximum production of withanolide A was shown in ammonia-nitrate ratio of 0.00/18.80 mM	[95]
	R1000/A4	Transformation efficiency 64.2% with R1000 and 38.8% with A4. Cocultivation medium was supplemented with acetosyringone (100 *µ*M)	[90]

Shoot	LBA 9402	Root cultures synthesized various withanolides. Withanolide D was isolated and identified. Yield of withanolide D was almost 7-fold higher than control	[114]

## Abbreviations

BAP: 6-Benzylaminopurine
2,4-D: 2,4-Dichlorophenoxyacetic acid
KN: Kinetin
IAA: Indole-3-acetic acid
IBA: Indole-3-butyric acid
NAA: 1-Naphthalene acetic acid
TDZ: Thidiazuron
TIBA: 2,3,5-Triiodobenzoic acid
GA: Gibberellic acid
MeJ: Methyl jasmonate
SA: Salicylic acid.
